# A primary-school-based study to reduce the prevalence of childhood obesity – the EdAl (Educació en Alimentació) study: a randomized controlled trial

**DOI:** 10.1186/1745-6215-15-58

**Published:** 2014-02-14

**Authors:** Lucia Tarro, Elisabet Llauradó, Rosa Albaladejo, David Moriña, Victoria Arija, Rosa Solà, Montse Giralt

**Affiliations:** 1 Unit of Farmacobiology, Facultat de Medicina i Ciències de la Salut, Universitat Rovira i Virgili, C/Sant Llorenç 21, Reus 43201, Spain; 2 Health Education and Promotion, Facultat de Medicina i Ciències de la Salut, Universitat Rovira i Virgili, C/Sant Llorenç 21, Reus 43201, Spain; 3 Technological Center of Nutrition and Health (CTNS) - TECNIO - URV – CEICS Avda Universitat 1, Reus 43204, Spain; 4 Unitat de Bioestadística, Facultat de Medicina, Universitat Autònoma de Barcelona, Avda de Can Domènech, Cerdanyola del Vallès 08193, Barcelona, Spain; 5 Unit of Nutritional Epidemiology, IISPV, Institut d’Investigació en Atenció Primària, Jordi Gol i Gorina, Facultat de Medicina i Ciències de la Salut, Universitat Rovira i Virgili, C/Sant Llorenç 21, Reus 43201, Spain; 6 Unit of Lipids and Arteriosclerosis Research, CIBERDEM, Hospital Universitari Sant Joan, IISPV, Facultat de Medicina i Ciències de la Salut, Universitat Rovira i Virgili, C/Sant Llorenç 21, Reus 43201, Spain

**Keywords:** Childhood obesity, Healthy lifestyle, Obesity prevention, Intervention program, Health promoting agent

## Abstract

**Background:**

Obesity is one of the main determinants of avoidable disease burden.

To implement a program by university students acting as “health promoting agents” (HPAs) and to evaluate the effects on obesity prevalence of the primary-school-based program that promotes healthy lifestyle, including dietary and physical activity recommendations over 28 months.

**Methods:**

Two school clusters were randomly assigned to intervention (24 schools, 1,222 pupils) or control (14 schools, 717 pupils); 78% of pupils were Western European. Mean age (±SD) was 8.4 ± 0.6 years (49.9% females) at baseline. Generalized linear mixed models were used to analyze differences in primary outcome between both groups. Data collected included body mass index (BMI) every year. Dietary habits and lifestyle questionnaires were filled in by the parents at baseline and at the end of the study. The interventions focused on eight lifestyle topics covered in 12 activities (1 hour/activity/session) implemented by HPAs over 3 school academic years.

**Results:**

At 28 months, obesity prevalence in boys was decreased −2.36% in the intervention group (from 9.59% to 7.23%) and increased 2.03% (from 7.40% to 9.43%) in the control group; the difference was 4.39% (95% CI 3.48 to 5.30; *P* = 0.01). The boys in the intervention group had an effective reduction of −0.24 units in the change of BMI z-score (from 0.01 to −0.04), compared to control (from −0.10 to 0.09); 5.1% more intervention pupils undertook physical activity >5 hours/week than control pupils (*P* = 0.02).

Fish consumption was a protector (odds ratio 0.39; 95% CI 0.23 to 0.67) while “fast-food” consumption was a risk factor for childhood obesity (odds ratio: 2.27; 95% CI 1.08 to 4.77).

**Conclusions:**

Our school-based program, conducted by HPA students, successfully reduced childhood obesity prevalence in boys.

**Trial registration:**

International Standard Randomized Controlled Trial Number: ISRCTN29247645.

## Background

Obesity is one of the main determinants of avoidable disease burden [1-4]. The adverse effects of obesity on health status are not fully reversible and so a stronger focus on the prevention of obesity has been advocated [5]. Since overweight status and obesity in adulthood are predicated on childhood and adolescent weight, obesity prevention should start early in life [4].

Treatment to decrease childhood obesity, addressing different areas and focusing on behavioral changes towards healthier lifestyles, has been a means of reducing morbidity and mortality from non-communicable diseases [6]. Lifestyle has become increasingly sedentary. Playing console games and watching television [7] have dramatically replaced physical activity and participation in organized sports [8]. The consequence is a relationship between overweight and television viewing hours as part of a sedentary lifestyle. However, non-school computer use and reading were not part of this relationship [9-11].

One important target group is the child of school-age, especially when old enough to understand and young enough to be influenced (that is, the age-group around pre-adolescence) [12]. Schools are excellent learning environments where children spend a great deal of time [13]. Some school-based interventions focused on 6- to 12-year-old children have shown some beneficial effects on overweight prevalence, or percentage obesity [14,15]. A recent review of interventions for the prevention of obesity in children found strong evidence to support beneficial effects of obesity prevention programs on body mass index (BMI), specifically those programs targeting children aged 6 to 12 years [16].

Intervention duration, preferably 3 years, and parental involvement as a primary target of intervention, can help to reduce obesity [17]. However, the causes of failure of school intervention to prevent obesity still need investigating [4,18]. Waters and colleagues [16] suggested that it may no longer be justified to test short-term (3 months to 1 year), behaviorally-focused school-based interventions for 6- to 12-year-old children.

Our study (EdAl; *Educacio en Alimentacio*; Education in Alimentation) is a program that can reach most school children in our population [19]. Routinely, teachers have many tasks to perform and, perhaps, are too busy with new educational challenges. An alternative we proposed was to involve young students from medical and health science departments of the local university who, as part of their new curriculum, receive health education oriented towards school-based interventions. Hence, we designed the EdAl program with defined experimental activities to be conducted by university students given specific instruction aimed at training them to become “health promoting agents” (HPAs) as part of their undergraduate science/medical curriculum.

A recent meta-analysis recommends that research be encouraged to test innovative interventions which exploit new technologies, behavioral theories, and methodologies, including system science [20]. Hence, we proposed a program for the prevention of childhood obesity by implementing healthy lifestyle choices. The innovative design involved the use of university students as HPAs in local schools.

Our hypothesis was that a regular, systematic, educational intervention in primary school improves lifestyle choices and reduces obesity. The interventions were based on eight nutritional and physical activity objectives.

The aims of the study were: 1) to design a health promotion program for implementation by HPAs in primary schools; and 2) to evaluate the effects of a 3-year school-based program of lifestyle improvement, including diet and physical activity over a period of 28 months, on the prevalence of obesity.

## Methods

The protocols, rationale, randomization, and techniques of study have been published in *Trials*[19]. This study was approved by the Clinical Research Ethical Committee of the Hospital Universitari Sant Joan of Reus, Universitat Rovira i Virgili (Catalan ethical committee registry #20; ref: 08-07-24/07aclproj1). The protocol conformed to the Helsinki Declaration and Good Clinical Practice guides of the International Conference of Harmonization. This study followed the CONSORT criteria (see the Additional file 1).

### University program

The training and standardization of educational intervention activities to promote healthy lifestyles in schools were carried out over two university courses (basis of health education and behavior; strategy design, implementation, health program evaluations) carried out in the same university academic year for undergraduates from medical and health-science departments, and included training and standardization for each activity as well as the implementation of these activities in schools (45 hours per academic course as described in [19]) leading to the title of HPA. These two courses are taught in each of the three university undergraduate basic-science academic years. HPA students implemented the activities for which they had been training and standardizing in the second course. Hence, the university HPA in the first year of training implemented, in the first school academic year, the four activities focusing on four of the eight lifestyle topics. The following year, the second university HPA group developed four new training and standardization activities focusing on the other four of the eight lifestyle topics for implementation in the second school academic year. The third university HPA group implemented, in the third school academic year, four new training and standardization activities to reinforce the previous eight lifestyle topics.

### Study population

The coordinating center in Reus developed a randomization scheme in which the schools in Reus were designated as Group A (intervention) and the schools in the three other towns of Cambrils, Salou and Vila-seca were designated as Group B (control). The socio-demographic indicators in all the towns surrounding Reus were similar. Children attending the schools in both groups (intervention and control) live in close proximity within each school’s catchment area. Hence, after randomization, Reus schools were chosen for intervention, while the surrounding schools were the control population to avoid crossover of intervention details.

The research team arranged to meet all the schools in the proposed intervention group (Reus) and, following the explanation of the objectives, each school decided whether or not to take part in the study. The directors of the EdAl program explained the sequence of events of the EdAl activities and, subsequently, the EdAl coordinator arranged a meeting with every school that opted to participate. Intervention institutions were 24 schools involving 36 classrooms and 1,550 pupils in Reus. Control institutions consisted of 14 schools involving 39 classrooms and 800 pupils in the three surrounding towns of Cambrils, Salou and Vila-seca. The imbalance in enrollment was due to the high number of schools in the intervention group that were keen and enthusiastic to enter the EdAl program; we did not place any limit on the number of schools that wished to participate.

All strategies focused on children between 7 and 8 years of age. The program targeted the whole school community, including parents, pupils, staff and teachers. To be representative of the child population, the schools selected needed to have at least 50% of the children in the classrooms volunteer to participate. We offered the program to all schools, whether public (funded by the government and termed “charter” schools) or private.

Inclusion criteria were name, gender, date, place of birth and parental consent. These data were registered at the start of the program, while weight, height, body mass index, and waist circumference variable (identified set of anthropometric measures) were recorded in each of the 3 years. For logistic reasons, the first group of school children was enrolled in 2006 (children born in 1998–1999) and followed-up for 3 school academic years (2006–2009). A second group of school children was enrolled in 2007 (children born in 2000–2001) and also followed-up for 3 school academic years. Hence, the measurements were performed each school academic year between the years 2006 and 2010. In the intervention group, all children of the selected classrooms were exposed to the intervention, and the control group did not receive any type of intervention. The data were collected on all children, but only the data from individuals who provided written informed consent signed by parents or guardians in each academic year were included in the final analyses.

Questionnaires regarding eating habits (Krece Plus) developed by Serra Majem and colleagues [21], and physical activity, level of parental education and their lifestyles (AVall) developed by Llargués and colleagues [22] were filled in by the parents at baseline and at the end of the study.

### Intervention program

The intervention program consisted of three components: 1) classroom practice by the HPA to highlight eight healthy lifestyle habits [23], termed educational intervention activities; 2) teaching practice by the HPA using specially-designed booklets (as teaching aids) which focused on the same lifestyle topics presented as educational activities; 3) parental activities to be included with that of their children.

The educational intervention activities focused on eight lifestyle topics based on scientific evidence [23] to improve nutritional food item choices (and avoidance of some foods), healthy habits such as teeth-brushing and hand-washing and, overall, adoption of activities that encourage physical activity (walking to school, playground games) and to avoid sedentary behavior [23].

Each of the eight topics was integrated within educational intervention activities of 1 hour/activity, prepared and standardized by the HPAs and then implemented in children’s classrooms. In the first school academic year, we focused on four topics: 1) to improve healthy lifestyle; 2) to encourage healthy drinks intake (and avoidance of unhealthy carbonated/sugared beverages); 3) to increase vegetables and legumes consumption; and 4) to decrease candies and pastries while increasing the intake of fresh fruits and nuts. These corresponded to four standardized activities (1 hour/activity). In the second year, the remaining four of the eight selected lifestyle topics were addressed: 5) to improve healthy habits within a set timetable (home meals, teeth-brushing, hand-washing) and physical activity participation; 6) to increase fruit intake; 7) to improve dairy product consumption; and 8) to increase fish consumption. These corresponded to four standardized activities. Finally, in the third school academic year, four standardized activities were introduced that reinforced the eight lifestyle topics implemented in the previous 2 academic years. Thus, the intervention program was based on eight lifestyle topics incorporated in 12 activities which were disseminated over 12 sessions (1 hour/activity/session), and prepared, standardized and implemented as four activities per school academic year by the HPAs in the school classrooms. The activities or sessions were implemented every 2 weeks over a 2-month period, each academic year. All 12 activities or sessions were conducted over a period of 28 months (3 school academic years).

The educational intervention activity as a classroom practice consisted of three components: 1) experimental development of activities relating to healthy lifestyle habits using food-item selection (free food items provided by local producers) for the children to experience the organoleptic quality of the items which may, or may not, be new to them; 2) assessment of activity performed in classroom; and 3) activities developed for use at home.

Teaching practice used specific booklets designed to address the same lifestyle topics as the educational intervention activities (see Additional file 2). The booklets (teaching aids) were also employed by the regular school teacher over the 28 months of the program.

Another aspect of the intervention program was to involve parents in activities with their children. This intervention for parents was the same educational nutritional activities that were directed towards the children by the HPA. The intention was to have parents and their children interact in the healthy nutrition and lifestyle choices.

### Outcomes

Primary outcomes included overall prevalence of obesity, as well as prevalence segregated by gender, according to the International Obesity Task Force [24] recommendations for better international comparisons of data. Secondary outcomes were changes (overall and segregated by gender) in measures of adiposity such as BMI z-score and waist circumference, and incidence and remission of excess weight (overweight + obesity), as well as changes in lifestyles such as eating habits and physical activity. Weight, height and waist circumference were obtained as described previously [19]. The prevalence of underweight was analyzed according to Cole and colleagues [25] using 17 kg/m^2^ as the cut-off point. BMI z-score was analyzed according to the World Health Organization Global InfoBase [26].

### Statistical analyses

Descriptive data are presented as means ± SD or percentages (and 95% CI). General linear mixed models were used to analyze differences between the intervention and control pupils with respect to the prevalence of obesity.

We estimated that, with a sample of 700 pupils per group, the study would have 83.5% power to detect a difference of 5 percentage points (range: expected 9% of obesity prevalence in intervention group and 14% in control group) between the intervention and control schools, with respect to the primary outcome (prevalence of obesity).

Anthropometric measurements were conducted at baseline when the pupils were second-third graders (7 or 8 year olds), the following year, and at the end of the study when they were fourth-fifth graders (10 or 11 year olds). Lifestyle questionnaires were filled in at baseline and the end of the third year of the study.

The numbers of subjects with any specific dietary habit were expressed as percentages of the total number of individuals being evaluated. In order to evaluate risk and protective factors for childhood obesity, logistic regression analyses were performed at baseline, with no distinction between control group and intervention group. The odds ratio (ORs) and the 95% CI were calculated for dietary patterns and lifestyles based on the Krece Plus Questionnaire [21] and the AVall Questionnaire [22], respectively. The main analyses were performed with the modified intention-to-treat population (that is, subjects with at least one post-baseline measurement of weight and height). The analysis did not use any imputation missing method, the implication being that missing data were random.

## Results

### University program

There were 60 HPAs enrolled in the program to conduct the 12 standardized activities.

### Enrollment

**Figure 1:**
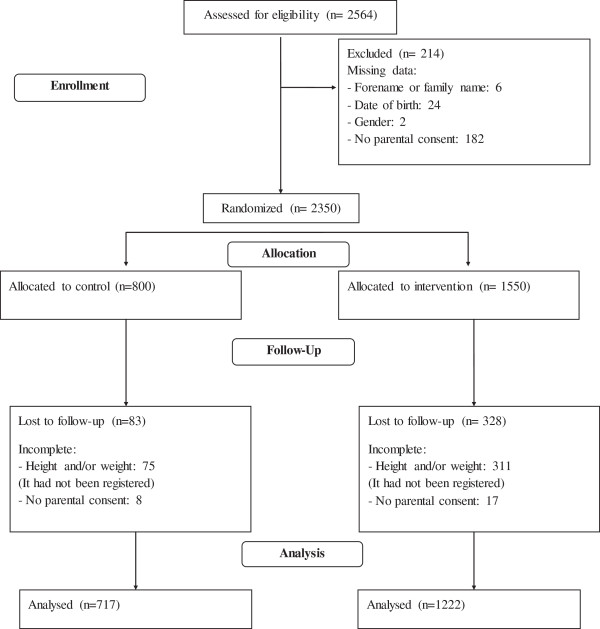
**Flow of subjects through the study.** Incomplete height and/or weight (measured in the second and/or third academic year) and no parental consent signed (second or third academic year) were two criteria that included the participants who moved to another school that was not taking part in the *Educacio en Alimentacio* (EdAl) study.

Figure 1 shows the recruitment and flow of pupils in the intervention and control groups over the course of the study. The modified intention-to-treat population in the intervention group and the control group were 1,222 and 717 pupils, respectively. The rate of parental consent was 92%.

Tables 1 and 2 summarize the baseline characteristics of study participants. BMI, calculated as weight in kilograms divided by the square of the height in meters (mean ± SD), was 17.69 ± 3.19 kg/m^2^ in the intervention group and 17.09 ± 2.93 kg/m^2^ in the control group, while the medians (P25 to P75) were 16.90 (15.50 to 19.22) and 16.37 (15.15 to 18.40), respectively.

**Table 1:** Baseline anthropometric characteristics of pupils: intervention group and control group

	**Intervention group (n = 1,222)**			**Control group (n = 717)**		
	**Mean ± SD**			**Mean ± SD**		
	**Boys**	**Girls**	**Total**	**Boys**	**Girls**	**Total**
Weight (kg)	31.26 ± 7.11	30.71 ± 7.26	30.99 ± 7.19	29.3 ± 6.75	29.53 ± 7.23	29.42 ± 7
Body mass index (kg/m^2^)	17.73 ± 3.16	17.65 ± 3.22	17.69 ± 3.19	17 ± 2.77	17.17 ± 3.07	17.09 ± 2.93
Height (m)	132.3 ± 6.27	131.4 ± 6.53	131.9 ± 6.41	130.7 ± 6.73	130.4 ± 6.82	130.6 ± 6.78
Fat mass (kg)	6.44 ± 4.49	6.67 ± 4.15	6.55 ± 4.33	5.43 ± 3.42	6.47 ± 4.92	5.97 ± 4.3
Lean mass (kg)	24.71 ± 3.62	23.76 ± 3.09	24.26 ± 3.41	23.89 ± 3.97	23.31 ± 3.42	23.59 ± 3.7
Waist circumference (cm)	60.69 ± 7.85	58.98 ± 7.33	59.85 ± 7.64	59.8 ± 7.18	60.04 ± 8.28	59.93 ± 7.77

Body mass index was calculated as weight (kg)/height squared (m^2^). Fat and lean mass were calculated using a Standard Beam Balance (Tanita TBF-300 Body Composition Analyzer; Tanita Corporation of America, Inc. 2625 South Clearbrook Drive, Arlington Heights, Illinois 60005, USA).

**Table 2:** Baseline characteristics of pupils: intervention group and control group

	**Intervention group (n = 1,222)**	**Control group (n = 717)**
	**Total (%)**	**Total (%)**
**Ethnicity**		
Western European	76.2	80.4
Eastern European^a^	4.2	4.3
Latin American	7.4	10.7
North African (Arab origin)	10.6	2.7
Sub-Sahara African	0.4	0.1
East Asian (Chinese origin)	1.1	1.3
Indian	0.1	0.4
North American	0.0	0.1
**Breastfeeding**^**b**^		
0–1 month	66.5	65.2
1–3 months	7.9	6.9
3–6 months	12.5	12.7
>6 months	13.1	15.3
**Father’s education level**^**b**^		
Less than high school	3.3	1.2
High school	42.3	36.5
Technical training	36.5	38.9
Non-university higher education	1.3	1.4
University degree	16.6	22.1
**Mother’s education level**^**b**^		
Less than high school	3.1	1.6
High school	38.7	31.6
Technical training	37.1	39.6
Non-university higher education	1.1	2.2
University degree	20.1	24.9

^a^Eastern European includes Russia and newly independent states (former USSR). ^b^Data on maternal breastfeeding were solicited as this could be a possible confounding variable, as was the level of parental education.

Table 2 also contains breastfeeding characteristics and parental education levels, which were similar in both groups.

### Attrition rate

Figure 1 shows the recruitment and retention of pupils in the intervention and control schools. Among the 2,350 pupils assessed at the beginning of the second or third grade, 1,939 (82.5%) pupils (89.6% of those allocated to the control group and 78.8% of those allocated to the intervention group) were reassessed when they were in the fifth or sixth grade, and valid measurements were obtained. Drop-outs in both groups are understood to be missing at random.

### Primary outcome: prevalence of obesity

At 28 months, there was a significant difference of −4.39% in obesity prevalence in boys between the intervention and control groups (*P* = 0.02; that is, reduction from the first to the third year in the intervention group of −2.36% (9.59% to 7.23%) while in the control group this increased by 2.03% (7.40% to 9.43%) (Table 3)).

**Table 3:** Baseline and end of study measurements of categorized body mass indices in the intervention and control groups

**Body mass index classification**			**Baseline (% (n))**	**End of study (% (n))**	**Change (%)**	**Baseline vs end of study ( *P *** **value)**	**Intervention vs control ( *P *** **value)**
Underweight	Intervention	Boys	1.65 (10)	1.20 (8)	−0.45	0.636	0.768
		Girls	0.69 (4)	1.42 (9)	0.73	0.270	0.014
		Total	1.18 (14)	1.31 (17)	0.13	0.857	0.030
	Control	Boys	0.89 (3)	0.34 (1)	−0.55	0.627	
		Girls	2.43 (9)	1.87 (6)	−0.56	0.795	
		Total	1.69 (12)	1.13 (7)	−0.56	0.490	
Normal weight	Intervention	Boys	66.78 (404)	67.47 (449)	0.69	0.811	<0.001
		Girls	70.81 (410)	71.36 (451)	0.55	0.849	0.385
		Total	68.75 (814)	69.37 (899)	0.62	0.761	<0.001
	Control	Boys	80.77 (273)	67.00 (199)	−13.77	< 0.001	
		Girls	72.43 (268)	69.78 (224)	−2.65	0.450	
		Total	76.41 (541)	68.45 (423)	−7.96	0.0013	
Overweight	Intervention	Boys	21.98 (133)	24.10 (160)	2.12	0.386	0.001
		Girls	20.03 (116)	20.41 (129)	0.38	0.886	0.292
		Total	21.03 (249)	22.30 (289)	1.27	0.465	0.001
	Control	Boys	10.95 (37)	23.23 (69)	12.28	< 0.001	
		Girls	17.57 (65)	21.81 (70)	4.24	0.178	
		Total	14.41 (102)	22.49 (139)	8.08	< 0.001	
Obese	Intervention	Boys	9.59 (58)	7.23 (48)	−2.36	0.155	0.016
		Girls	8.46 (49)	6.80 (43)	−1.66	0.280	0.762
		Total	9.04 (107)	7.02 (91)	−2.02	0.075	0.047
	Control	Boys	7.40 (25)	9.43 (28)	2.03	0.390	
		Girls	7.57 (28)	6.54 (21)	−1.03	0.657	
		Total	7.49 (53)	7.93 (49)	0.44	0.836	

Prevalence of obesity and overweight were categorized using the cut-off criteria proposed by the International Obesity Task Force [24]. Prevalence of underweight was analyzed according to Cole *et al*. [25] using 17 kg/m^2^ as the cut-off point. Categorical outcomes were analyzed using generalized linear models. Analyses were performed with the modified intention-to-treat analysis.

### Secondary outcomes

#### BMI z-score, waist circumference, incidence and remission of excess weight (overweight plus obesity)

At 28 months of the study, BMI z-score was significantly lower in the intervention group compared to controls (overall: −0.03 vs 0.01, *P* < 0.001; in boys: −0.04 vs 0.09, *P* < 0.001; and in girls: −0.01 vs −0.03, *P* < 0.001). For pre- versus post-intervention, the BMI z-score increase was significant only in boys in the control group (−0.10 to 0.09, *P* = 0.015; Table 4). Waist circumference changed significantly between the first and third year of the study in the intervention and control groups (*P* = 0.043).

**Table 4:** **Secondary outcomes** (**body mass index z-score, waist circumference and body mass index) at baseline and at the end of the study in the intervention and control groups**

			**Baseline (mean (95% CI))**	**End of study (mean (95% CI))**	**Baseline vs end of study ( *P *** **value)**	**Intervention vs control ( *P *** **value)**
Body mass index z-score	Intervention	Boys	0.01 (−0.07, 0.10)	−0.04 (−0.11, 0.04)	0.367	<0.001
		Girls	0.00 (−0.08, 0.08)	−0.01 (−0.09, 0.06)	0.755	<0.001
		Total	0.05 (−0.01, 0.11)	−0.03 (−0.08, 0.03)	0.388	<0.001
	Control	Boys	−0.10 (−0.20, 0.00)	0.09 (−0.03, 0.21)	0.015	
		Girls	−0.02 (−0.12, 0.08)	−0.03 (−0.14, 0.08)	0.941	
		Total	−0.11 (−0.18, -0.04)	0.01 (−0.07, 0.09)	0.105	
Waist circumference (cm)	Intervention	Boys	60.69 (60.07, 61.30)	67.44 (66.73, 68.16)	<0.001	0.269
		Girls	58.98 (58.39, 59.57)	65.96 (65.24, 66.67)	<0.001	0.108
		Total	59.85 (59.42, 60.28)	66.72 (66.21, 67.22)	<0.001	0.043
	Control	Boys	59.80 (59.04, 60.57)	66.39 (65.39, 67.38)	<0.001	
		Girls	60.04 (59.20, 60.88)	66.10 (65.10, 67.10)	<0.001	
		Total	59.93 (59.36, 60.50)	66.24 (65.54, 66.94)	<0.001	
Body mass index (kg/m^2^)	Intervention	Boys	17.73 (17.48, 17.97)	18.86 (18.60, 19.13)	<0.001	0.442
		Girls	17.65 (17.39, 17.91)	18.76 (18.49, 19.04)	<0.001	0.596
		Total	17.69 (17.51, 17.87)	18.82 (18.63, 19.01)	<0.001	0.381
	Control	Boys	17.00 (16.71, 17.30)	18.48 (18.10, 18.86)	<0.001	
		Girls	17.17 (16.85, 17.48)	18.28 (17.92, 18.64)	<0.001	
		Total	17.09 (16.87, 17.30)	18.38 (18.12, 18.64)	0.001	

Continuous outcomes were analyzed through mixed models of repeated measures. Analyses were performed with the modified intention-to-treat analysis.

At 28 months, BMI was not statistically different in the intervention and control groups (*P* = 0.381).

The incidence of excess weight was significantly higher in the control group (51/414, 12.3%, *P* = 0.021) than in the intervention group (57/709, 8%), particularly in boys in the control group (35/207, 16.9%, *P* = 0.011) compared to boys in the intervention group (33/352, 9.4%). Girls did not present significant differences between the control and intervention groups. Remission of excess weight was not significantly different between the intervention and control groups, nor in relation to gender.

#### Lifestyle evaluation (including eating habits and physical activity)

Tables 5 and 6 summarize dietary and lifestyle habits, including time spent doing physical exercise, watching television and playing video games and other leisure-time activities. In the intervention group, the percentage of pupils having a cereal breakfast (68.3% vs 72.9%; *P* = 0.01), a second fruit per day (38.3% vs 42.6%, *P* = 0.03), at least one vegetable a day (68.9% vs 72.6, *P* = 0.04), and vegetables more than once a day (25.7% vs 32.0%, *P* = 0.001) increased. Conversely, the percentage of legumes consumed more than once a week decreased in the control group (76.9% vs 70.9%, *P* = 0.01).

**Table 5:** Food habits assessed at baseline and at the end of the study in the intervention and control groups

	**Intervention group**			**Control group**			
	**Baseline (%)**	**End of study (%)**	***P*** **Value**	**Baseline (%)**	**End of study (%)**	***P*** **value**	**Intervention vs control ( *P *** **value)**
**Krece plus questionnaire**							
Breakfast	99.2	98.2	0.11	99.8	99.2	0.18	0.50
Dairy product at breakfast	91.9	92.3	0.72	95.4	93.0	0.08	0.09
Cereals at breakfast	68.3	72.9	0.01	74.8	74.5	0.89	0.13
Pastry at breakfast	17.5	15.3	0.17	16.2	15.1	0.62	0.70
Daily fruit or natural juice	70.9	72.9	0.30	78.1	78.9	0.95	0.59
Fruit, second per day	38.3	42.6	0.03	38.4	37.6	0.78	0.14
Dairy product, second per day	81.9	80.6	0.43	85.9	84.2	0.4	0.81
Vegetables, daily	68.9	72.6	0.04	72.7	73.8	0.67	0.42
Vegetables, >1 per day	25.7	32.0	0.001	23.2	26.9	0.13	0.50
Fish, regularly	73.9	74.8	0.65	76.10	74.3	0.4	0.35
Fast food, >1 per week	7.9	8.9	0.41	7.9	8.7	0.63	0.92
Legumes >1 per week	75.5	75.2	0.85	76.9	70.9	0.01	0.06
Candy >1 per day	12.9	12.0	0.52	11.6	9.9	0.32	0.68
Pasta or rice, daily	57.3	57.9	0.80	59.9	62.2	0.37	0.58
Cooking with olive oil at home	96.6	97.4	0.32	97.1	97.9	0.38	0.85
**AVall questionnaire**							
*Before leaving home*							
Dairy products	84.3	82.4	0.20	86.6	83.8	0.15	0.65
Pastry	3.9	2.4	0.005	2.8	2.6	0.15	0.50
Cereals	37.6	38.2	0.87	38.4	39.7	0.83	0.94
Fresh fruit or natural juice	20.9	20.6	0.07	18.8	18.5	0.21	0.93
Sandwich	23.9	25.2	0.45	19.1	22.1	0.93	0.69
Juice packaged/soft drinks	9.6	7.2	0.93	8.4	8.9	0.32	0.46
*Break (Midmorning)*							
Dairy products	17.3	12.7	0.79	13.7	10.1	0.29	0.51
Pastry	3.8	1.7	<0.001	4.1	2.2	0.002	0.95
Cereals	6.5	5.6	0.68	5.2	4.7	0.45	0.71
Fresh fruit or natural juice	12.5	12.7	0.59	12.2	15.5	0.05	0.18
Sandwich	45.5	49.2	0.09	43.3	48.3	0.08	0.71
Juice packaged/soft drinks	11.4	6.1	0.67	9.9	12.8	0.48	0.41

The parent or guardian completed a self-reported form at school, which included Krece Plus Questionnaire regarding nutritional habits [21] and AVall Questionnaire regarding lifestyles [22]; this included food items consumed at breakfast (before leaving home and midmorning). This questionnaire was filled in by parents twice during the study (at baseline and at the end of the study).

**Table 6:** Physical activity and leisure activities assessed at baseline and at the end of the study in the intervention and control groups

	**Intervention group**			**Control group**			
	**Baseline (%)**	**End of study (%)**	***P*** **value**	**Baseline (%)**	**End of study (%)**	***P*** **value**	**Intervention vs control ( *P *** **value)**
**Television and/or video games**							
0–1 hours/day	7.9	6.8		8.2	9.4		
1–2 hours/day	42.4	40.5		46.0	42.8		
2–3 hours/day	33.3	34.1		33.3	32.1		
3–4 hours/day	10.4	12.7		7.7	11.1		
4–5 hours/day	3.9	3.5		3.8	3.4		
>5 hours/day	1.9	2.5	0.06	1.1	1.3	0.39	0.59
**After-school physical activity**							
0–1 hours/week	24.9	18.8		21.2	20.8		
1–2 hours/week	9.3	11.4		10.2	9.6		
2–3 hours/week	25.2	20.8		24.4	22.1		
3–4 hours/week	17.9	16.4		19.5	18.8		
4–5 hours/week	11.2	12.9		11.0	14.1		
>5 hours/week	11.4	19.7	<0.001	13.2	14.6	0.21	0.02
**Leisure-time activity**							
Park or garden, daily	61.4	62.7	0.63	61.7	64.6	0.24	0.52
Park or garden, weekend	52.9	52.6	0.94	44.2	45.9	0.44	0.51
Sport with father >3 hours/week	29.5	35.1	<0.001	34.4	36.5	0.01	0.47
Sport with mother (>3 hours/week	22.1	26.1	<0.001	26.3	28.9	0.06	0.43

The AVall Questionnaire is about lifestyles, and includes hours of television watched, physical activity after school and leisure-time activities [22]. This questionnaire was filled in by parents twice during the study (at baseline and at the end of the study).

Physical activity change (from baseline to 28 months) analyzed by gender showed that the percentage of pupils that perform >5 hours/week physical activity in the intervention group were: boys 14.9 to 24.1%, P < 0.001; girls 8.2 to 15.5%, P = 0.005. In the control group the corresponding values were: boys 17.4 to 17.8%, P = 0.860; girls 9.6 to 11.9%, P = 0.804. There were no differences between groups in relation to gender.

Of these intervention pupils, more boys involved themselves in >5 hours/week physical activity than did the girls (24.1% vs 15.5%, *P* = 0.001), but this gender difference did not exist in the control group.

In the intervention group, the percentage of pupils consuming pastry before setting off for school decreased from 3.9% to 2.4% (*P* = 0.005), and in the mid-morning break decreased from 3.8% to 1.7% (*P* < 0.001). In the control group, the percentage of pupils consuming pastries in the mid-morning break also decreased (4.1% vs 2.2%, *P* = 0.002) while the consumption of fruit or natural juice increased (12.2% vs 15.5%, *P* = 0.05). There were no significant differences between groups with respect to other nutritional habits.

Furthermore, in the intervention group at the end of study, a higher percentage of boys than girls consumed dairy products for breakfast (94.1% vs 90.5%, *P* = 0.038) including a second dairy product daily (84.1% vs 77.4%, *P* = 0.010). The only differences between genders we observed were in relation to these two aspects of dairy products consumption.

It is interesting to note that the percentage of mothers dedicating >3 hours/week to sporting activities increased in the intervention group (22.1% vs 26.1%, *P* < 0.001).

We observed a larger reduction of BMI z-score in children who performed >4 hours/week physical activity (−0.07), whereas BMI z-score reduction was less in children who performed <4 hours/week physical activity (−0.03). This indicates that children who perform more hours of physical activity per week have a tendency to reduce their BMI z-score, albeit without achieving statistical significance in our present study (*P* = 0.171).

A lower prevalence of obesity of 3.9% was associated with a higher level of maternal education. A higher prevalence of 11.5% was associated with a lower educational level (*P* < 0.01). The same trend was observed in relation to paternal education level, although the differences were less statistically evident. In summary, after 28 months of the EdAl intervention the children’s parents increased their involvement with their children’s activities to >3 hours/week (29.5% vs 35.1%, *P* < 0.001 for fathers; 22.1% vs 26.1%, *P* <0.001 for mothers), whereas in controls the increase in physical activity occurred only in fathers (34.4% vs 36.5%, *P* = 0.01) (Table 6). No significant differences were observed between groups with respect to hours per day devoted to television and/or video games.

### Impact of some additional factors on obesity

At baseline with one obese parent, the child obesity prevalence was 9.06% while, with neither parent being obese, the prevalence was 5.91%. However, there were no significant differences between those children with, and those without, obese parents (*P* = 0.214) in relation to the intervention outcomes.

**Figure 2:**
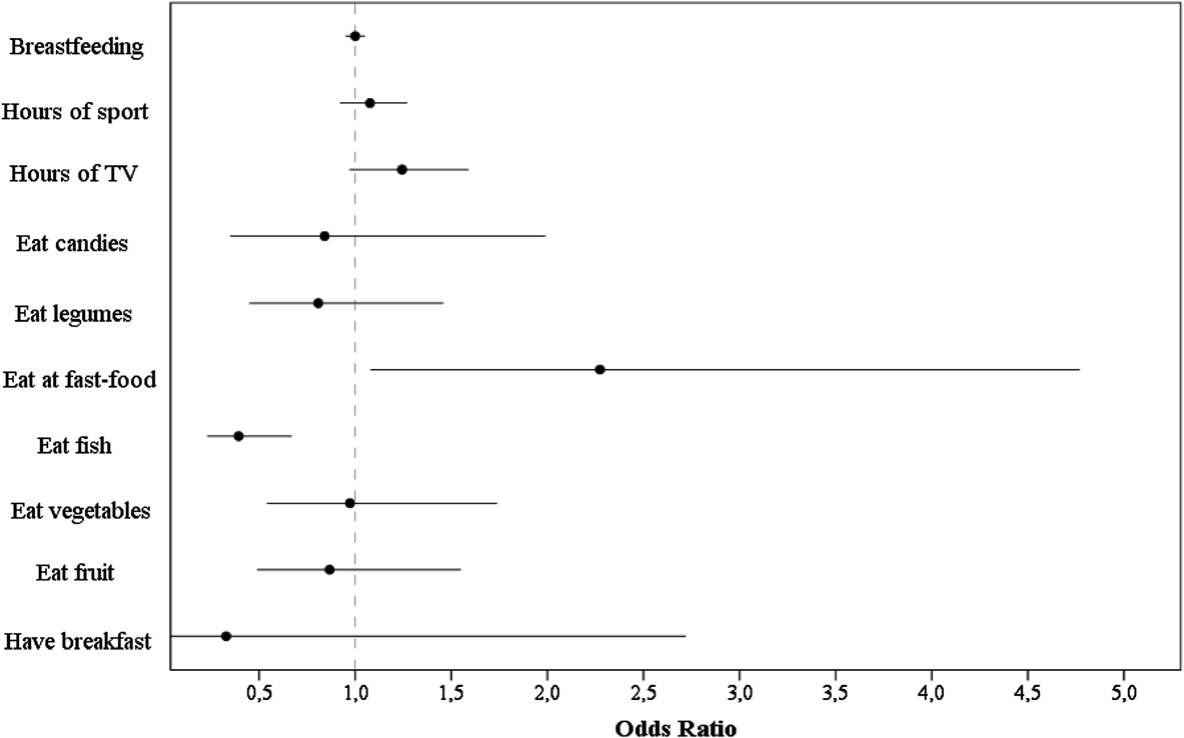
**Obesity prevalence risk factors.** These risk factors are listed in order of appearance in the questionnaires: breastfeeding, sporting activity time, hours watching television (TV) or video games, sweetened snacks and beverages, consumption of legumes, fast-food, fish, vegetables, fruit and having breakfast. The results showed that eating fish is a protector against childhood obesity while eating fast-food is a risk factor. These risk factors are measured as odds ratios.

Figure 2 summarizes the ORs of obesity related to some of the more relevant dietary habits. For example, fish consumption was found to be a protective factor against obesity whereas fast-food consumption ≥1/week increased the risk of obesity.

### Adverse events

Adverse events were not systematically collected in the questionnaire. However, no such effects were reported by the children or their parents/guardians.

## Discussion

The hypothesis of our study was that an intervention focusing on lifestyle improvement that included diet and physical activity can reduce the prevalence of obesity in children. Our study sample is representative of current Spanish society with respect to economic, social and ethnic distributions. Our hypothesis was partially fulfilled in that the data showed a reduction in prevalence of obesity of 4.39% in boys, but not in girls. There were also significantly greater reductions in BMI z-scores in boys of the intervention group compared to the control group of boys. Waters and colleagues [16] stated that an intervention may be effective in reducing, relative to the change in the control group, the size of the change in BMI z-score pre- to post-intervention by −0.15 units. Specifically, a change of −0.20 units in total and −0.24 units in boys participating in the EdAl intervention reveals that the intervention was effective. However, BMI and waist circumference measurement were not statistically different between groups at 28 months. Further, the intervention group had less new cases of excess weight than the control group. This highlights the efficacy of our program in preventing (or reducing) childhood obesity.

Some studies, such as the AVall study [27], have shown BMI not to be reduced despite changes in lifestyle, albeit the intervention group having a lower increase in BMI than the control group. However, the control group also showed changes in lifestyle and physical activity. Nevertheless, the prevalence of overweight and obesity increased less in the intervention group than in control group [27,28]. Kriemler and colleagues [29] proposed applying a multi-component program, including increased physical activity, over the period of 1 school year. The target outcomes were physical and psychological health in young school children around 7 years of age. The results indicated a reduction in adiposity but not BMI; obesity *per se* had not been evaluated [29].

Schools have been proposed as the ideal places to conduct obesity prevention programs, to prevent weight gain or to reduce the prevalence of overweight and obesity [20]. Half of the published school-based interventions reported statistically significant beneficial effects compared with the control in at least some of the body-weight-related indices. These included BMI, BMI z-score, overweight and obesity prevalence, waist circumference, skinfold thickness, and percentage body fat [20].

The study by Wang and Lobstein [30] predicted 0.5% increase/year in childhood obesity prevalence in Mediterranean Europe. In our control group, the rate of obesity was shown to rise from 7.4% in year 1 to 9.4% in year 3 of the study; an increase of 2% in 3 years (that is, greater than the 1.5% predicted by Wang and Lobstein). Conversely, in the intervention group, obesity was reduced from 9.6% in year 1 to 7.2% in year 3 of the study, a reduction of 2.4% in obesity prevalence. At 3 years of intervention, obesity in the girls was reduced by −1.7%, and by −1.0% in the control group (that is, no statistically significant difference between the two groups). Furthermore, at baseline, the subgroup of obese and overweight represents 30.07% in the intervention group and 21.9% in the control group while, at the end of the study, the obese and overweight subgroup represents 29.32% and 30.42%, respectively (that is, in the intervention group the proportion remained almost the same while, in the control group, this proportion was increased by almost 8.5%).

The duration of the intervention program to reduce overweight and obesity is another factor that can influence the outcomes [16]. The present study suggests that a notable decrease in the prevalence of obesity and improvement in lifestyle in children to avoid overweight and obesity can be achieved in 28 months (that is, 3 school academic years).

In our study, the percentage obesity in girls remained relatively unchanged during the study. Physiological differences between boys and girls at around 10 to 11 years of age could explain these findings. Certainly, the differences in age-of-onset of puberty between girls and boys could explain some of the variation in BMI response observed in our study. Gortmaker and colleagues [31] observed obesity prevalence changes in girls, but not in boys; however, Planet Health had a duration of 2 years in which educational sessions were incorporated within the curricula by primary school teachers [31]. The present study had a duration of 3 years and the educational sessions were not part of the curriculum [19]. As outcomes, the incidence was different between the group of boys in EdAl, but not in Planet Health. On the other hand, Planet Health presented differences in girls in terms of remission, while the EdAl study does not. Thus, while the EdAl study has prevented childhood obesity cases, Planet Health has reduced the initial cases of obesity, both interventions inducing positive benefits in the intervention group of children. The findings of differences in outcomes indicate that, since mediators of anthropometric changes are different between males and females, future interventions need to be specifically tailored to gender [32].

We used the International Obesity Task Force [24] criteria for better international comparisons of data. The prevalence of obesity at baseline in our study was 9.0% in the intervention group, and 7.5% in the control group. The EnKid Study by Serra Majem and colleagues [21] in northeast Spain (conducted between 1998 and 2000) indicated 9.8% obesity at the same age as the participants in the present study. In Spain, using the same obesity definition criteria [24] as the present study, measurements made in 2012 by Sánchez-Cruz and colleagues showed 25.3% as overweight and 9.6% obesity prevalence in children between 8 and 13 years of age [33]. As such, the obesity prevalence in 2012 in the Sánchez-Cruz study is similar to the control groups of both genders and also girls in the intervention group in the EdAl study, whereas the boys in the intervention group of the present study showed the lowest obesity prevalence.

The observed efficacy of our intervention, with respect to BMI-related outcomes, may be due to our approach that focused on the nutritional quality of food intake, and included increasing the physical activity of the pupils in conjunction with their parents. At 28 months of intervention, an increase of up to 19.7% of children dedicated >5 hours/week to extra-curricular physical activities. The percentage of parents participating in the children’s sporting activities of >3 hours/week also increased, and has led to a lifestyle change that has become characteristic of our intervention program.

The tendency towards a reduction in BMI z-score when children perform >4 hours/week physical activity needs to be confirmed in other long-term studies.

Our results showed that fish consumption was a protective factor against childhood obesity. A high proportion of fish in the diet is characteristic of the Mediterranean diet, but which children, in general, do not find very palatable. Recently, results from a study conducted in 1,764 healthy children and adolescents aged 6 to 19 years attending Seventh Day Adventist schools in the USA [34] indicated that the frequencies of consumption of grains, nuts, vegetables and low nutrient-dense foods were inversely related to the risk of being overweight, while dairy products increased the risk.

In the present study, weekly consumption of fast-food was identified as a risk factor for childhood obesity, despite the low percentage (<9%) of our pupils who consumed ≥1 fast-food items per week. A study involving 6,740 school-aged children aged 7 to 18 years in Xi’an in China indicated that sedentary lifestyles that included watching television, playing video games, and using computers are risk factors for obesity [35].

The Kiel Obesity Prevention Study showed that children from high Socio Economic Status families had some favorable, and sustained, effects with respect to BMI changes over an 8-year study period [36]. Furthermore, the PANACEA study concluded that parents’ academic level was inversely associated with overweight and obesity prevalence, together with better adherence to the Mediterranean diet [37]. As such, these articles follow the same direction of trends as our current study. Nevertheless, long-term studies would be necessary to evaluate whether parental education levels have an influence on sustained effects post-intervention.

More specific investigation is required to establish the factors underlying obesity in girls at this age. The type of intervention that would be effective across the childhood population is eagerly sought. The cost-effectiveness of our type of obesity-prevention program is associated with social involvement by a public university. The feasibility of our program is based on offering course credits within the academic curriculum as an inducement for HPAs to participate in these educational interventions in schools. The dissemination of this program depends on selecting personnel who can undertake these activities in children. In a well-constructed program, the economic cost of obesity is reduced while social interaction between generations of young people is improved.

Concerning the limitation of the study in the school setting, there are possible sources of “contamination” of the control group. For example, if a school in the control group acquired some information deployed in the intervention group as a result of chatting among parents and school friends, this can motivate control students and families to adopt the intervention recommendations and, thus, they do not behave as controls should. Further, we believe that lifestyles and physical activity measured by self-reporting (parents of children) provide only a limited validity of these measures. Hence, it is necessary to assess the feasibility, effectiveness, and sustainability of this intervention study, as well as its reproducibility in the context of other schools, before the results can be generalized.

## Conclusions

In conclusion, our primary-school-based program performed by HPAs reduces, within 28 months, the prevalence of obesity in boys by 4.39%, but not in girls.

## Abbreviations

BMI: body mass index; EdAl: *Educacio en Alimentacio*; (Education in Alimentation); HPA: health promoting agent; OR: odds ratios.

## Competing interests

The authors declare that they have no competing interests.

## Authors’ contributions

MG, RA, LT, VA and RS designed the research (project, conception, development of overall research plan, and study oversight). MG, RA, LT, EL and RS conducted the research (hands-on conduct of experiments and data collection). RA, LT, MG, EL, VA and RS provided essential reagents, or provided essential specialist materials (applies to authors who contributed by providing constructs and databases necessary for the research). DM analyzed data and performed statistical analysis. RS, LT, EL, MG, and DM wrote the manuscript (authors who made a major contribution). The final manuscript was read and approved by all co-authors. RS and MG had primary responsibility for the study, and manuscript content. All authors read and approve the final manuscript.

## Supplementary Material

Additional file 1 CONSORT criteria was considered for this study. Click here for file

Additional file 2 Booklets designed for teachers to address the same lifestyle topics as the educational intervention activities to scholars. Click here for file
