# Supplementary material for: A primary-school-based study to reduce the prevalence of childhood obesity – the EdAl (Educació en Alimentació) study: a randomized controlled trial
Source: Trials. 2014 Feb 14;15:58. doi: 10.1186/1745-6215-15-58 (PMC3926975; doi:10.1186/1745-6215-15-58)
Supplement: Additional file 2 — Booklets designed for teachers to address the same lifestyle topics as the educational intervention activities to scholars. [file 1745-6215-15-58-S2.pdf]

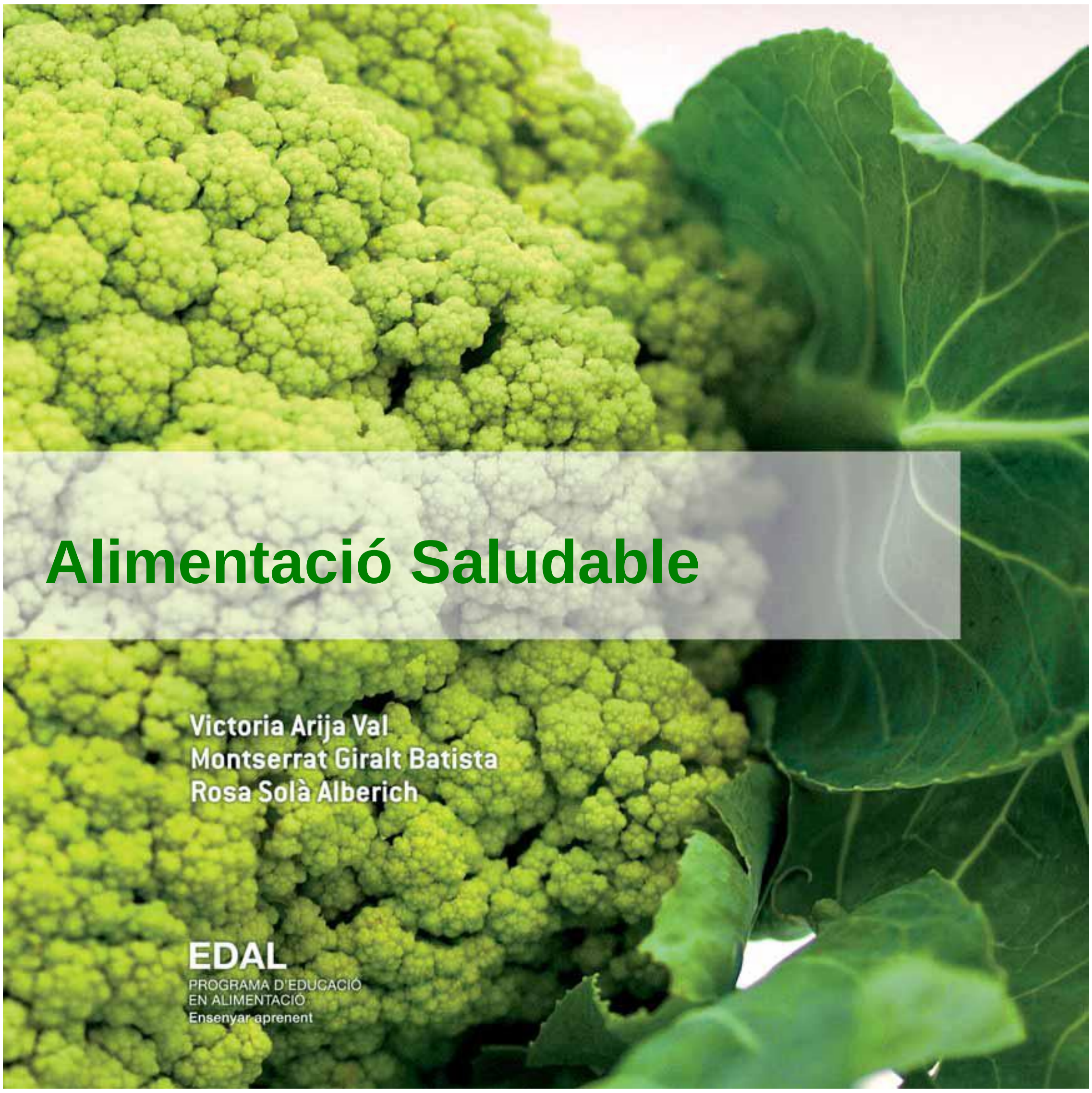

Title: Healthy diet

This book offers an approach to the theme of feeding, nutrition and lifestyles and it is addressed to parents with 7-8 years old children or primary school teachers.

This book focuses on educate to parents and primary teachers about how is a healthy diet and it promote a healthy lifestyles. The book introduces the relation between diet and healthy and which are the main nutritional risk factors in our population. Also, introduces the concept of Mediterranean diet.

The book exposes knowledgably the principal food groups, while it explains about all of them: nutritional content, servings recommended, and which foods belong to each food group.

Afterwards, it explains how to carry out a healthy and balanced diet, giving different advices to be able to follow it. And finally, it gives some examples about healthy daily menus; and it explains The EDAL project.

Table of contents

1. Relationship between diet and health.

2. Challenges of nutritional education. Strategies to promote healthy lifestyles: feeding.

3. Fundaments of healthy feed.

3.1 Food groups:

3.1.1 Protein food.

3.1.2 Dairy products.

3.1.3 Fruits.

3.1.4 Vegetables.

3.1.5 Cereals and potatoes.

3.1.6 Oils, fats and nuts.

3.1.7 Water.

3.2 Superfluous food groups.

3.2.1 Sweets and sweetened beverages.

3.2.2 Ice-creams and pastry.

3.2.3 Alcoholic beverages.

4. How do we do an optimal feeding, healthy and balanced?

4.1 The variety.

4.2 The quantity.

4.3 The daily and weekly distribution.

5. To make the healthy and balanced food menus.

6. EDAL Program: Education and promotion of healthy lifestyles and dietary habits.

Book details

ISBN:

Division:

Edit: Fundació Privada Reddis, Reus (Spain)

Pub Date: JAN 2007

Pages: 71

Copyright:

Edition: 1

Format: Paperback

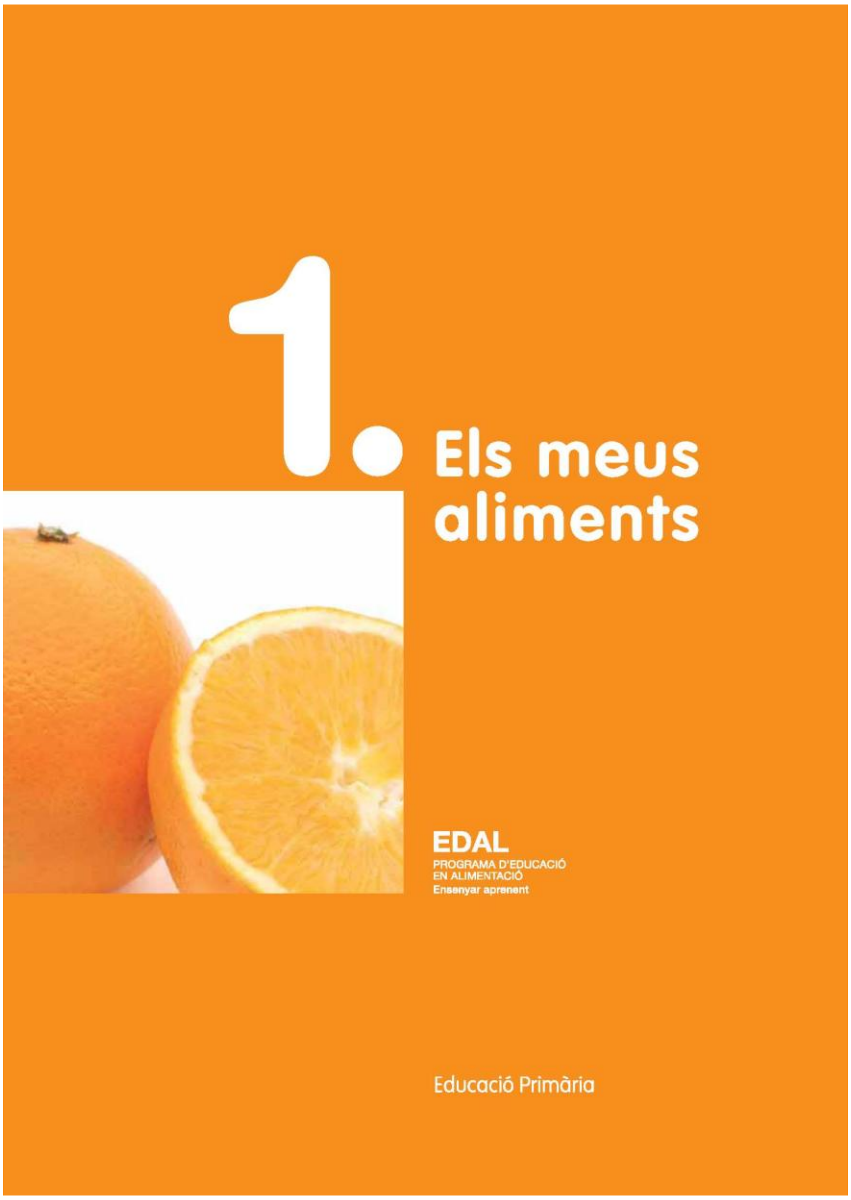

Title: My foods (Workbook 1)

This book offers an approach to the theme of feeding, nutrition and lifestyles focus on children between 7-8 years old. This book offers some activities and exercises that children should be able to do as they get to learn and grow, and it divided in 4 lifestyle and nutritional topics that children need to know and learn, based on evidence. Type of the activities are questions about what is their favorite food, their ideal food menu, their favorite flavors, so it is achieved that children thing and talk about food preferences and it try to taste new flavors. Also, this book uses riddles, songs, proverbs and recipes.

Thus, it becomes more enjoyable for the children, and also, it is closely linked to culture, country habits and lifestyles and typical food.

Table of contents

1. Personal data: the child can explain his/her personality, his/her anthropometric measures (weight, height), his/her name, age, the milk tooth, and his/her academic course.

2. Taste.

3. Healthy drinks.

4. Vegetables and legumes.

5. Pastries and candies.

Book details

ISBN:
Division:
Edit: Fundació Privada Reddis, Reus (Spain)
Contribute: Diputació de Tarragona (Spain)
Pub Date: JAN 2007
Pages: 48

Copyright:
Edition: 2
Format: Paperback

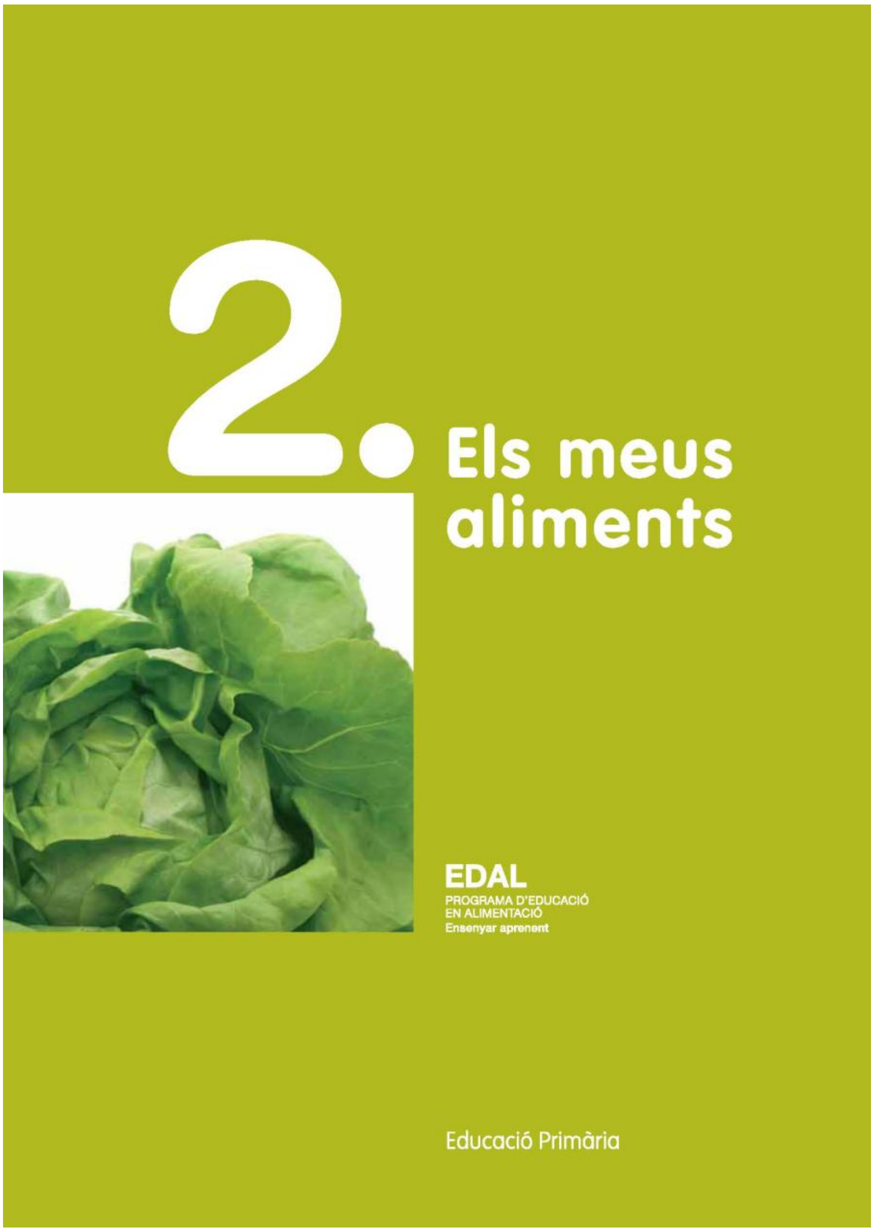

Title: My foods (Workbook 2)

This book offers an approach to the theme of feeding, nutrition and lifestyles focus on children between 9-10 years old.

This book offers some activities and exercises that children should be able to do as they get to learn and grow. However, the level of the concepts presented in this book it's higher than in Workbook 1. It divided in 4 lifestyle and nutritional topics more, that children need to know and learn, based on evidence. These complement Workbook 1.

Type of the activities, is the same as in the previous Workbook, are questions about what is their favorite food, their ideal food menu, their favorite flavors, so it is achieved that children thing and talk about food preferences and it try to taste new flavors. Also, this book uses riddles, songs, proverbs and recipes. Thus, it becomes more enjoyable for the children, and also, it is closely linked to culture, country habits and lifestyles and typical food.

Table of contents

1. Personal data: the child can explain his/her personality, his/her anthropometric measures (weight, height), his/her name, age, the milk tooth, and his/her academic course.

2. Timetable and healthy habits.

3. Fruits.

4. Dairy products.

5. Fish.

Book details

ISBN:
Division:
Edit: Fundació Privada Reddis, Reus (Spain)
Contribute: Diputació de Tarragona (Spain)
Pub Date: JAN 2007
Pages: 48

Copyright:
Edition: 2
Format: Paperback

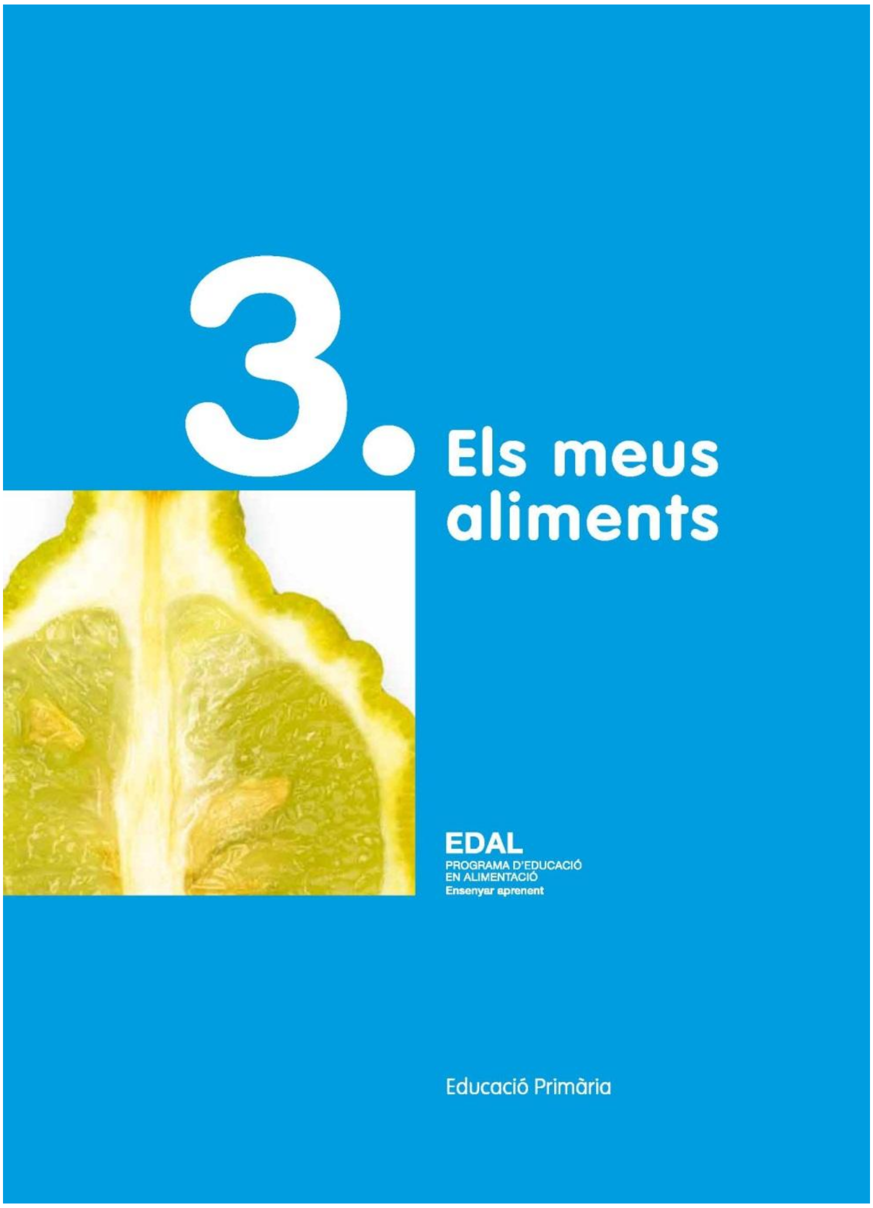

Title: My foods (Workbook 3)

This book offers an approach to the theme of feeding, nutrition and lifestyles focus on children between 10-12 years old. This book offers some activities and exercises that children should be able to do as they get to learn and grow, and it divided in 8 lifestyle and nutritional topics that it reinforcement of two previous Workbooks, but now with more difficulty. Type of the activities are composition a food daily menu, to be able to identify the different types of food, such has, different cheese types or fish types, the benefits to eat some different foods, to learn how can cooking different types of food.

Also, this book uses riddles, songs, proverbs and recipes. Thus, it becomes more enjoyable for the children, and also, it is closely linked to culture, country habits and lifestyles and typical food.

This workbook contains a CD-Room with the answers, nutritional information and didactic aims about exercises addressed to primary school teachers.

Table of contents

1. Timetable and nutritional habits.

2. Fruits (reinforce both workbook 1 and 2).

3. Vegetables and legumes (reinforce both workbook 1 and 2).

4. Dairy products (reinforce both workbook 1 and 2).

5. Fish (reinforce both workbook 1 and 2).

6. Healthy drinks (reinforce both workbook 1 and 2).

7. Candies and snacks.

8. Healthy lifestyles (reinforce both workbook 1 and 2).

Book details

ISBN:
Division:
Edit: Fundació Privada Reddis, Reus (Spain)
Contribute: Diputació de Tarragona (Spain), Caixa Manresa i “La Caixa”.
Pub Date: MAR 2010
Pages: 96

Copyright:
Edition: 1
Format: Paperback and CD-Room
